# Clinical and Epidemiological Investigation of TCM Syndromes of Patients with Coronary Heart Disease in China

**DOI:** 10.1155/2012/714517

**Published:** 2012-03-22

**Authors:** Yi Ren, Minzhou Zhang, Keji Chen, Shijie You, Jianjun Li, Liheng Guo, Lei Wang

**Affiliations:** ^1^Guangdong Provincial Hospital of TCM, 111 Dade Road, Guangdong, Guangzhou 510120, China; ^2^Xiyuan Hospital, China Academy of Chinese Medical Science, Beijing 100091, China; ^3^Fuwai Cardiovascular Disease Hospital, Chinese Academy of Medical Science, Beijing 100037, China

## Abstract

To compare the regional differences in TCM syndromes of patients with coronary heart disease (CHD) between North and South China. A total of 624 patients with a diagnosis of CHD, confirmed by coronary angiography, were included in the comparative analysis to determine the occurrence pattern, characteristics of TCM syndrome distribution, and differences in syndrome combinations and major syndrome types (deficiency or excess) between North and South China. The incidence of CHD tended to be higher in North China (54.6%) compared with that in South China (45.4%). The proportions of patients with a qi-deficiency syndrome (83.7%), turbid phlegm syndrome (68.9%), or blood stasis syndrome (91.5%) were generally higher in the South group, while the proportion of patients with a cold congelation syndrome (7.9%) was identified to be obviously higher in the North group (*P* < 0.01). Moreover, compared with that in the South group, the overall frequency of syndrome combinations tended to be lower in the North group (*P* < 0.01); and the most common types of TCM syndrome were excess syndrome (193, 56.6%) and primary deficiency and secondary excess syndrome (244, 86.2%) in the North and South groups, respectively (*P* < 0.01). A regional difference does exist in the TCM syndromes of patients with CHD between North and South China, indicating that the prevention and treatment of CHD in South China should not only focus on promoting blood circulation and removing blood stasis, but also include supplementing qi and eliminating phlegm

## 1. Introduction

Coronary heart disease (CHD) is the most common form of heart disease and remains to be a persistent public health burden worldwide. An epidemiological study showed that there were about 1,300,000 new cases of CHD diagnosed in China each year [1]. Previous studies have documented the efficacy and safety of Xiongshao capsule in reducing restenosis after percutaneous coronary intervention (PCI) in CHD patients [2, 3], indicating that traditional Chinese medicine (TCM) has potential advantages in the treatment of CHD. As an essential element of TCM theories, syndrome differentiation is the basis for the treatment of all diseases, including CHD. Therefore, it is important to characterize the TCM syndromes of patients with CHD; however, previous studies have not addressed the potential regional differences in TCM syndromes of these patients. In our study, we conducted a comparative analysis of 624 patients with a CHD diagnosis confirmed by coronary angiography to determine the characteristics and differences in TCM syndrome distribution between North and South China.

## 2. Materials and Methods

### 2.1. Study Design

This is a prospective observational study about the regional differences in TCM syndromes of patients with CHD between North and South China. Hospitalized patients with a CHD diagnosis confirmed by coronary angiography in Beijing, Fuwai Cardiovascular Hospital and Guangdong Provincial Hospital of TCM from December 2007 to December 2010 were included in the study.

### 2.2. Inclusion/Exclusion Criteria

Male or female patients diagnosed as CHD and confirmed by coronary angiography were eligible for inclusion. The diagnosis of CHD was made according to the “nomenclature and criteria for diagnosis of ischemic heart disease. Report of the Joint International Society and Federation of Cardiology/World Health Organization task force on standardization of clinical nomenclature” [4]. And the diagnostic criteria for TCM syndrome differentiation was based on the “criteria for TCM syndrome differentiation of patients with coronary heart disease” (revised in 1990), released by China Society of Integrated Traditional Chinese and Western Medicine [5].

 All of the procedures of coronary angiography were performed by highly skilled physicians using Judkins approach. The findings of coronary angiography were interpreted by at least two experienced readers, and the final diagnosis of CHD is made according to the angiography report.

Patients complicated with other diseases that may interfere with the syndrome differentiation of this study, or those suffering from other serious diseases of major organs or infective diseases were exclude from the study. And those who cannot or not willing to complete the study; or those who had psychiatric disorders or intellectual dysfunctions were also excluded.

### 2.3. Syndrome Differentiation

**Figure 1:**
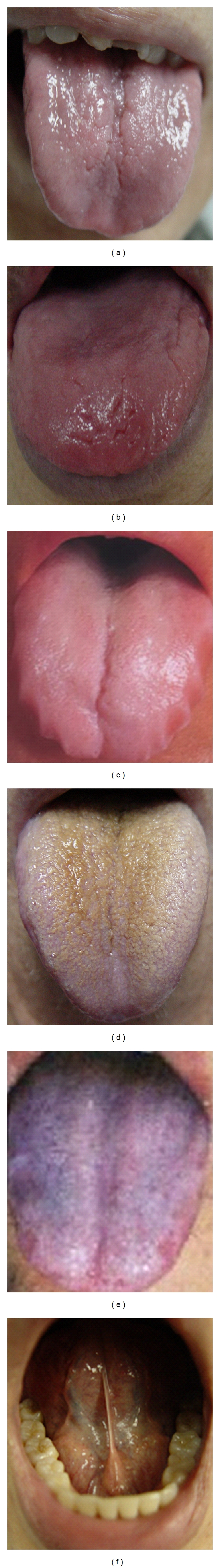
Typical tongue manifestations in the study. Notes: (a) common tongue manifestation of yang deficiency syndrome; (b) common tongue manifestation of yin deficiency syndrome; (c) common tongue manifestation of Qi deficiency syndrome; (d) common tongue manifestation of turbid phlegm syndrome; (e) and (f) common tongue manifestation of blood stasis syndrome.

The TCM syndrome differentiation was performed on the day of admission, which was based on the information gathered from inspection, auscultation, olfaction, inquiring, and palpation of the pulse. This study collected information on the tongue with a unified type of digital camera, and pictures shown in Figure 1 represented some typical tongue manifestations. Pulse manifestations and other diagnostic information were determined independently by two experienced cardiovascular physicians with attending doctor title to ensure objective evaluation. The 7 basic syndromes of patients with CHD included Qi-deficiency syndrome, yin-deficiency syndrome, yang-deficiency syndrome (including yang Qi vacuity desertion), Qi stagnation syndrome, blood stasis syndrome, cold congelation syndrome, and turbid phlegm syndrome (including phlegm heat).

### 2.4. Statistical Analysis

The statistical analysis of this study was performed by using SPSS18.0 software. An independent sample *t*-test was conducted for between group comparisons of quantitative data, which were represented as $\overset{̅}{x}\pm s$; the Wilcoxon-rank sum test was applied for nonnormal data and unequal variances. The qualitative data were analyzed by contingency table *χ* ^2^ test, and the significance level was set at *α* = 0.05.

## 3. Results

### 3.1. General Characteristics

A total of 624 patients with a CHD diagnosis confirmed by coronary angiography were included in the study, 351 from Beijing Fuwai Cardiovascular Hospital and 273 from Guangdong Provincial Hospital of TCM. Of these patients, 472 (75.6%) were male and 152 (24.4%) were female, with an average age of 60.2 ± 11.8 years. The diagnosis of CHD could be classified into stable angina (109 and 17.4%), unstable angina (202 and 32.4%), and acute myocardial infarction (313 and 50.2%). The most common co-morbidities included hypertension (373 and 59.8%), diabetes (159 and 25.5%), hyperlipemia (229 and 36.7%), and cerebrovascular disease (44 and 7.1%).

### 3.2. Regional Distribution of CHD Patients

The division of North or South China was based on the provinces that patients come from (see Table 1), with the Tsinling Mountains—Huai River as the dividing line, north of the Tsinling Mountains—Huai River as North China while south of the Tsinling Mountains—Huai River as South China. It was shown that the incidence of CHD tended to be higher in North China compared with that in South China (see Table 2).

### 3.3. North-South Differences in TCM Syndromes

The presence or absence of each of the 7 TCM syndrome types, considered as a binary variable, was compared between the North group and the South group. Results showed that the proportions of patients with a Qi-deficiency syndrome, turbid phlegm syndrome, or blood stasis syndrome were generally higher in the South group, while the proportion of patients with a cold congelation syndrome was identified to be obviously higher in the North group (*P* < 0.01, see Table 3). No significant differences were noted between the two groups in terms of the proportions of patients with a yin-deficiency syndrome, yang-deficiency syndrome, or Qi stagnation syndrome (*P* > 0.05, see Table 3).

Moreover, the trends of syndrome combinations as well as the proportions of major syndrome types (deficiency or excess) were also compared between the two groups. Results revealed that, compared with that in the South group, the overall frequency of syndrome combinations tended to be lower in the North group (*P* < 0.01, see Table 4); the most common types of major TCM syndromes were excess syndrome (193, 56.6%) and primary deficiency and secondary excess syndrome (244, 86.2%) in the North and South groups, respectively (*P* < 0.01, see Table 4).

## 4. Discussion

The incidence of CHD varies substantially around the world. Chambless et al. [6] reported that in 18 (e.g., Finland and England, etc.) out of the 29 MONICA populations the incidence of acute myocardial infarction or possible coronary events was higher than 400/100,000, of which North Karelia, Finland had the highest rate (818/100,000) and Beijing, China had the lowest (79/100,000). The incidence of CHD not only varies across different countries, but also across different regions even in a same country. Wielgosz and Lynne [7] reported that the mortality of CHD in white Americans aged 35–74 years old varied among different regions and cities across the United States, of which the northeast region had the highest mortality followed by the middle-west and south regions, and the west region had the lowest rate with about 2.5-fold difference among them. The results of the Sino-MONICA project [8] conducted in 12 monitoring areas revealed that Beijing, Hebei, Neimenggu, Anshan Liaoning, Heilongjiang, and Xinjiang had a male incidence of CHD ≥50/100,000; Shenyang, Liaoning, and Jinlin had an incidence between 25/100,000–50/100,000; while Shanghai, Jiangsu, and other regions in South China all had an incidence lower than 15/100,000, indicating a generally higher incidence of CHD in North China compared with that in South China. In the 624 patients with a CHD diagnosis confirmed by coronary angiography included in our study, with the Tsinling Mountains—Huai River as the dividing line, the proportions of patients from North China and South China were 54.6% and 45.4%, respectively. The incidence trend of CHD identified in our study was generally consistent with that in the Sino-MONICA project.

The investigation of inspection, auscultation and olfaction, inquiring and palpation of the pulse manifestations have great significance to analyze Chinese medicine pathogenesis of coronary heart disease and its treatment based on syndrome differentiation [9]. Previous studies on TCM syndromes of CHD were generally limited to regional observation in small areas, which could not represent the distribution characteristics of TCM syndromes of CHD patients in China. Due to the differences in environment, climate, diet, and body constitution, potential regional difference may exist in the TCM syndromes of CHD patients. Previous studies showed that, the most common TCM syndrome of CHD patients was blood stasis (81.4%), followed by Qi deficiency (56.8%), turbid phlegm (48.5%), and Yin deficiency (25.1%) in Beijing/Tianjin (North China) [10], while in Changsha (South China), the commonly seen syndromes included heart-blood stagnation, phlegm blocking heart vessel, cold congelating heart vessel, Qi stagnating heart vessel, Qi deficiency of heart, Yang deficiency of heart, Yin deficiency of heart [11], which was generally consistent with the findings of our study. Previous studies have not investigated the distribution of TCM syndromes of CHD. In this study, we chose Beijing Fuwai Cardiovascular Hospital and Guangdong Provincial Hospital of TCM as the study sites because the former is located in North China while the latter in South China; both sites are leading cardiovascular centers with a great number of CHD patients from North and South China, respectively. Thus, based on these two sites, it is possible to characterize the distribution of TCM syndromes of CHD patients in China.

Our study found that the proportions of patients with a Qi deficiency syndrome, turbid phlegm syndrome, or blood stasis syndrome were generally higher in South China, while the proportion of patients with a cold congelation syndrome was identified to be higher in North China. In terms of syndrome combinations, the observed frequencies of the 4 patterns in North China were two-syndrome combination > single syndrome > three-syndrome combination > four-syndrome combination, while those in South China were three-syndrome combination > two-syndrome combination > four-syndrome combination > single syndrome. Overall, the number of combined syndromes in North China tended to be fewer than that in South China. As for the deficiency or excess properties of TCM syndromes, excess syndrome was the most common type in North China, while primary deficiency and secondary excess syndrome had the highest rate in South China. These results could be explained by the following two reasons: on one hand, the environmental factors have contributed to the North-South differences. The Lingnan region is located within the subtropical zone characterized by hot and humid climate throughout the year, which is likely to consume Qi and injury Yin; and people there like cold food and cold environments, which may result in spleen and stomach injuries, as well as phlegm-damp retention. Therefore, turbid phlegm and Qi deficiency are common in patients from the Lingnan region. Whereas the weather in the northern region is much colder, which may cause congelation and stagnation; the attack of cold pathogens may result in impeded circulation of the blood and Qi, manifested by the symptom of angina, for this reason cold congelation syndrome is common in patients from North China. On the other hand, constitution factors also have an impact on the incidence trend of CHD. People from North China are relatively strong, and the attack of various pathogens always results in stagnation of Qi activity and blood circulation, thus the single excess syndrome of Qi stagnation or blood stasis is common in North China; while primary deficiency and secondary excess syndrome has a higher incidence in South China.

## 5. Conclusion

It is concluded that a regional difference exists in the TCM syndromes of patients with CHD between North and South China, indicating that the prevention and treatment of CHD in South China should not only focus on promoting blood circulation and removing blood stasis, but also include supplementing Qi and eliminating phlegm.

## Figures and Tables

**Table 1:** Provincial distribution of the 624 patients with CHD.

	Beijing	Hebei	Shanxi	Guangdong	Henan	Shandong	Heilongjiang	Neimenggu	Anhui	Others
*N*	119	70	56	259	18	18	15	14	11	44
Percentages (%)	19.1	11.2	9.0	41.5	2.9	2.9	2.4	2.2	1.8	7.1

**Table 2:** North-South distribution of the 624 patients with CHD.

	North China	South China
*N*	341	283
Percentages (%)	54.6	45.4

**Table 3:** Comparison of the presence/absence of each of the 7 syndrome types between North and South China.

	Qi deficiency		Yang deficiency		Yin deficiency		Cold congelation		Qi stagnation		Blood stasis		Turbid phlegm	
	syndrome		syndrome		syndrome		syndrome		syndrome		syndrome		syndrome	
	NO	Yes	NO	Yes	NO	Yes	NO	Yes	NO	Yes	NO	Yes	NO	Yes
North China	236 (69.2%)	105 (30.8%)	318 (93.3%)	23 (6.7%)	291 (85.3%)	50 (14.7%)	314 (92.1%)	27 (7.9%)	316 (92.7%)	25 (7.3%)	115 (33.7%)	226 (66.3%)	189 (55.4%)	152 (44.6%)
South China	46 (16.3%)	237 (83.7%)	257 (90.8%)	26 (9.2%)	243 (85.9%)	40 (14.1%)	276 (97.5%)	7 (2.5%)	268 (94.7%)	15 (5.3%)	24 (8.5%)	259 (91.5%)	88 (31.1%)	195 (68.9%)
*χ* ^2^	175.10		1.28		0.04		8.90		1.06		56.92		37.09	
*P*	0.00		0.26		0.85		0.003		0.30		0.00		0.00	

**Table 4:** Comparison of the frequencies of syndrome combinations and deficiency or excess syndrome between North and South China.

	Syndrome combinations				Deficiency or excess syndrome		
	Single syndrome	Two-syndrome combination	Three-syndrome combination	Four-syndrome combination	Deficiency syndrome	Excess syndrome	Primary deficiency and secondary excess syndrome
NorthChina	130 (38.1%)	164 (48.1%)	38 (11.1%)	9 (2.7%)	45 (13.2%)	193 (56.6%)	103 (30.2%)
SouthChina	15 (5.3%)	68 (24.0%)	172 (60.8%)	28 (9.9%)	10 (3.5%)	29 (10.3%)	244 (86.2%)
*χ* ^2^	222.73					197.03	
*P*	0.00					0.00	
